# Social Drivers of Health, Management Bias, and Outcomes of Symptomatic Neonates With Tetralogy of Fallot

**DOI:** 10.1016/j.jacadv.2026.103161

**Published:** 2026-08-20

**Authors:** Flora Nuñez Gallegos, Shiraz A. Maskatia, Yun Zhang, Jeffrey D. Zampi, George T. Nicholson, Courtney McCracken, Christopher E. Mascio, Christopher J. Petit, Jennifer Romano, Dominic Zanaboni, Michael L. O’Byrne, Alekhya Nanduri, Jeannette Wong-Siegel, Mark A. Law, Courtney R. Alvis, Shabana Shahanavaz, Sarosh P. Batlivala, Sarah Speed, Jeffery J. Meadows, Mariam Taleb, Athar M. Qureshi, Hala Khan, R. Allen Ligon, Rachel Weber, Bryan H. Goldstein, Andrew C. Glatz, Keila N. Lopez

**Affiliations:** a University of California, San Francisco, California, USA; b Stanford University School of Medicine, Palo Alto, California, USA; c Columbia University, New York, New York, USA; d University of Michigan, Ann Arbor, Michigan, USA; e Vanderbilt University Medical Center, Nashville, Tennessee, USA; f Kaiser Permanente, Atlanta, Georgia, USA; g West Virginia University, Morgantown, West Virginia, USA; h University of Michigan, Ann Arbor, Michigan, USA; i The Children’s Hospital of Philadelphia, Philadelphia, Pennsylvania, USA; j Washington University, St. Louis, Missouri, USA; k The University of Alabama, Birmingham, Alabama, USA; l Cincinnati Children’s Hospital, Cincinnati, Ohio, USA; m Texas Children’s Hospital, Houston, Texas, USA; n Children's Healthcare of Atlanta, Emory University, Atlanta, Georgia, USA; o UPMC Children’s Hospital of Pittsburgh, Pittsburgh, Pennsylvania, USA; p Children’s Hospital Colorado, Aurora, Colorado, USA

**Keywords:** childhood opportunity index, congenital heart disease, driving distance, morbidity, social drivers of health

## Abstract

**Background:**

Advances in tetralogy of Fallot care have improved outcomes; however, sociodemographic disparities among infants with symptomatic tetralogy of Fallot requiring neonatal intervention (sTOF) remain incompletely characterized.

**Objectives:**

This study aimed to investigate sociodemographic and socioeconomic factors associated with management strategy and clinical outcomes in sTOF.

**Methods:**

We conducted a retrospective cohort study of neonates with sTOF undergoing intervention between January 2005 and November 2017 at 9 centers in the Congenital Cardiac Research Collaborative. The primary outcome was management strategy: primary complete repair vs staged repair. The primary predictor was the Childhood Opportunity Index, a neighborhood-level health composite measure. Secondary predictors included distance to cardiac center, maternal race-ethnicity, language, and insurance. Group differences were assessed using chi-square/Fisher tests with Holm adjustment, and multivariable models determined associations between predictors and outcomes.

**Results:**

Among 417 neonates, 180 (43%) underwent primary repair and 237 (57%) staged repair. Most were prenatally diagnosed (59%), non-Hispanic White (63%), and publicly insured (54%). In univariate analyses, Childhood Opportunity Index and maternal race-ethnicity were associated with management strategy; these associations attenuated after adjustment. Infants living >100 miles from a cardiac center had a higher hazard for mortality compared with those <50 miles (HR: 2.38; 95% CI: 1.07-5.30; *P* = 0.033). Maternal race-ethnicity and public insurance were associated with longer hospital stays and increased in-hospital complications.

**Conclusions:**

Sociodemographic factors, including geography, race-ethnicity, insurance, and neighborhood opportunity, are associated with management and outcomes in neonates with sTOF. Distance to care, maternal race-ethnicity, and insurance have notable impacts on these outcomes and warrant further assessment to ensure equitable decision-making management strategies.

As advances in congenital heart disease (CHD) care continue, increased attention has focused on disparities associated with sex, race-ethnicity, and socioeconomic status (SES).^1^^,^^2^ In prior studies, these social drivers of health (SDOH) were associated with worse outcomes in those with critical CHD requiring neonatal intervention.^3^^,^^4^ Symptomatic infants with tetralogy of Fallot (sTOF) have critical cyanosis, hypercyanotic episodes, or are ductal dependent and require ICU care with either surgical or transcatheter intervention in the neonatal period. The optimal management strategy for these infants is debated between either primary complete repair (PR) or staged repair (SR, involving initial catheter- or surgical-based palliation followed by later complete TOF repair). Research by the Congenital Cardiac Research Collaborative (CCRC) has previously demonstrated that SR is associated with lower early mortality and neonatal morbidity, but that PR is associated with lower cumulative morbidity and reinterventions^5^ and is associated with lower cumulative health care costs.^6^

There is significant practice variability in sTOF management, and social and structural factors can significantly complicate immediate and long-term patient outcomes. Disparities in SDOH may influence both management strategy and patient outcomes in sTOF. The Childhood Opportunity Index (COI) has been linked with various health care disparities in children with CHD.^7^^,^^8^ As a global measure of neighborhood opportunity, COI represents a holistic measure of the social context in which a family receives and interacts with the health care system. There are 29 indicators of neighborhood opportunity, and these intersect with individual-level social and economic factors, which have also been independently linked to disparities.^9^, ^10^, ^11^

Understanding whether SDOH, COI, and structural determinants of health influence provider decision-making in sTOF is crucial, as strategy selection during the neonatal period can have long-term implications.^5^^,^^12^ While neonates have not directly experienced their neighborhood environment, measures such as COI serve as proxies for family-level context, including access to resources, stability, and barriers to care. These factors may influence prenatal counseling, referral patterns, and provider decision-making at the time of initial management.

This study seeks to investigate the association between neighborhood COI, SDOH, choice of initial sTOF management strategy, mortality, and morbidity in infants with sTOF. The primary endpoint was the choice of initial management strategy (primary vs staged repair). Secondary endpoints included mortality and measures of morbidity, including total hospital length of stay and hospital complications. We hypothesized that lower individual and neighborhood SDOH factors are associated with differences in infant sTOF management and increased mortality and morbidity.

## Methods

### Patient population

We conducted an analysis of data collected as part of a retrospective multicenter cohort study of infants (described previously) who underwent PR or SR for sTOF at ≤30 days of age.^5^^,^^12^ We utilized clinical data, maternal demographics, and individual measures of SES from neonatal patients between January 1, 2005, and November 30, 2017, at the 9 CCRC centers (Supplemental Table 1) where infants had palliative or surgical TOF procedures. CCRC centers represent a geographically diverse sample of pediatric cardiac centers, with detailed patient data extracted from individual patients' electronic medical records, and each center entering deidentified data into a study-specific electronic database. Exclusion criteria included infants receiving procedures at older than 30 days of age or those with TOF and any of the following combinations: atrioventricular septal defect, absent pulmonary valve, and major aortopulmonary collateral arteries supplying pulmonary blood flow. This study was reviewed and approved by the Institutional Review Board (IRB) at Cincinnati Children’s Hospital, which acted as the single IRB for all participating sites. The IRB granted a waiver of the informed consent requirement. All participating centers and the data coordinating center operated under a formal data-use agreement, which outlined the terms for data handling and explicitly prohibited the sharing of patient-level information.

### Variables

#### Predictor

The COI provides a composite measure based on 29 neighborhood-level indicators of health across 3 domains: education, health/environment, and social/economic. Patient census tract data were obtained by entering the home address at the time of the initial sTOF intervention into the United States Census Bureau Geocoding Website.^13^ Once the 11-digit GEOID corresponding to the census tract was recorded, it was matched to the COI (diversitydatakids.org) 2.0 Data Set. Within each census tract, normalized national data for the overall COI score were recorded. The COI classifies neighborhoods into 5 nationally rank-ordered categories: very low, low, moderate, high, and very high. Given our sample size, these categories were collapsed a priori into 3 groups: very low/low (categories 1-2), moderate (category 3), and high/very high (categories 4-5), based on the prespecified national COI thresholds rather than the distribution of our study sample.^8^ This grouping strategy was chosen to ensure adequate group sizes for statistical modeling and improve interpretability and is consistent with approaches used in prior pediatric cardiology studies.^14^

#### Outcomes

The primary outcome was the chosen sTOF management strategy (PR or SR). Secondary outcomes included survival at the last available follow-up and the following measures of morbidity: hospital complications (occurring outside the 72-hour postprocedure window and until discharge) and total hospital length of stay (LOS). Hospital complications included stroke, bleeding, vascular injury, thrombus, arrhythmia requiring therapy, cardiac arrest, etc. (Supplemental Table 2), as described in prior CCRC studies.^5^ Total hospital LOS was made up of the LOS for the single PR or the sum of multiple procedural LOS associated with the SR. For infants undergoing PR, LOS encompassed a single hospitalization. For those undergoing SR, total LOS included their initial hospitalization (where a SR was initially performed) and any subsequent hospitalizations related to their definitive surgical repair.

### Covariates

#### Maternal

Individual-level SES factors included maternal race-ethnicity (categorized as mutually exclusive: non-Hispanic White, non-Hispanic Black, and Hispanic), maternal insurance (private, public, or other [including military, self-pay, or unknown]), and maternal primary language (English and Spanish). For maternal primary language, any responses recorded as “other” were recoded as missing. For the “other race-ethnicity” variable, any responses previously recorded as “other” were recoded as missing. Driving distance from the home address on record at the time of the index procedure to the primary cardiac care center was calculated retrospectively by each participating center between April and May 2023 using Google Map. The shortest driving distance (in miles) without route restrictions (eg, tolls or highways) was recorded for each patient. Driving distance was categorized a priori as <50, 50 to 100, and >100 miles to represent clinically meaningful gradients in geographic access and travel burden to specialized congenital cardiac care, consistent with prior literature.^15^

#### Infant

Patient characteristics were abstracted from the CCRC database, including sex, prematurity, known genetic syndromes, extracardiac anomalies, presence of a prenatal diagnosis, significant peri-operative characteristics, treatment center, and treatment strategy (PR or SR).^5^

### Statistical analysis

#### Descriptive analyses

Summary statistics were analyzed using descriptive methods, with frequencies and percentages reported for categorical variables. Chi-square tests or Fisher exact tests were used as appropriate based on expected cell counts. When overall tests for multilevel categorical variables were significant, pairwise comparisons were performed with Holm *P* value adjustment. Continuous variables with a symmetrical distribution are reported as mean (SD), while asymmetric continuous data are reported as median (IQR). Descriptive statistics are presented as counts and percentages for categorical variables, and as medians (25th-75th percentiles) for continuous variables.

#### Primary outcome: repair strategy

To evaluate associations between SDOH and repair strategy (PR vs SR), we fit multivariable logistic regression models and reported ORs with 95% CIs and *P* values. Models were then adjusted for prematurity, DiGeorge syndrome, center, anatomic diagnosis (antegrade pulmonary blood flow vs pulmonary atresia), and preintervention intubation. We previously evaluated the potential mediating role of these clinical factors using formal causal mediation analysis (PROC CAUSALMED, SAS version 9.4); however, none of the candidate mediators demonstrated a statistically significant indirect effect. Therefore, these variables were retained as covariates in the primary multivariable models.

#### Interaction analyses

We prespecified 2 interaction terms in the multivariable models: race-ethnicity × COI and driving distance × COI. These interactions were evaluated on the multiplicative scale. Global tests of interaction used type III joint Wald tests; global interaction *P* values are reported. For the race-ethnicity × COI model, we also present adjusted simple effects of COI within race-ethnicity strata. Simple-effect estimates are shown as ORs (95% CI) with *P* values.

#### Secondary outcomes

We used separate Cox proportional-hazards models to explore the effects of COI (primary exposure) on survival, adjusting for covariables including prematurity, DiGeorge syndrome, center, anatomical diagnosis, and intubation status. Death was treated as the event of interest. Patients who remained alive at the last documented follow-up were censored at that date. Results are presented as HRs with 95% CIs and *P* values. Proportional hazards assumptions were evaluated using Schoenfeld residual–based supremum tests.

We conducted exploratory analyses to determine the associations between each marker of SES status (race-ethnicity, primary language, insurance, and driving distance) and 2 secondary outcomes (total LOS and hospital complications). Multivariable linear regression models were calculated for each combination of SDOH and LOS. Multivariable logistic regression models were applied to the outcome of hospital complications (yes/no). In both models, we adjusted for covariables of prematurity, DiGeorge syndrome, center, anatomic diagnosis, and need for intubation. Because LOS was right-skewed, we fit linear regression models to log10-transformed LOS and reported exponentiated coefficients as LOS ratios along with 95% CIs and *P* values. Model residuals were evaluated and demonstrated no meaningful departures from normality. Because these analyses were exploratory, no correction for multiple comparisons was made.

Two-sided α = 0.050 defined statistical significance. All analyses were conducted using SAS version 9.4.

## Results

### Study population

A total of 572 neonates with sTOF who underwent either PR or SR were included in the primary CCRC sTOF study.^5^ Of the 572 patients in the initial sTOF cohort, patients whose addresses were not included in the COI database were excluded from our study, resulting in 417 eligible participants (73% of the initial cohort). Baseline patient characteristics, socioeconomic factors, peri-operative anatomic and clinical data, and center are summarized in Table 1. Additional clinical and anatomic characteristics of the study cohort, along with the distribution of initial palliative procedures in the SR group, are presented in Supplemental Table 3. Overall, among maternal and socioeconomic characteristics, maternal race-ethnicity and COI were significantly associated with SR or PR management selection (*P* = 0.018 and *P* = 0.004, respectively). Prematurity (<37 weeks, *P* = 0.018), presence of DiGeorge syndrome (*P* = 0.037), and preintervention intubation (*P* = 0.006) were also associated with repair strategy (Table 1). While management differed markedly across CCRC centers (*P* < 0.001), maternal primary language, driving distance, and insurance status did not differ significantly between management groups. To assess the potential for selection bias due to missing COI data, a sensitivity analysis compared baseline demographic and clinical characteristics, as well as clinical outcomes, between patients with and without available COI data (Supplemental Table 4).

**Table 1:** Characteristics of the Study Population

	Overall	Staged Repair	Primary Repair	*P* Value
Maternal characteristics				
Race-ethnicity				
Non-Hispanic White	215 (63.05)	116 (57.14)	99 (71.74)	0.018
Non-Hispanic Black	55 (16.13)	40 (19.70)	15 (10.87)	
Hispanic	71 (20.82)	47 (23.15)	24 (17.39)	
Primary language				
English	372 (92.54)	210 (90.52)	162 (95.29)	0.072
Spanish	30 (7.46)	22 (9.48)	8 (4.71)	
Socioeconomic characteristics				
Distance to primary heart center (miles)				
<50	191 (45.80)	108 (45.57)	83 (46.11)	0.953
50-100	126 (30.22)	73 (30.80)	53 (29.44)	
>100	100 (23.98)	56 (23.63)	44 (24.44)	
Insurance				
Private	168 (41.48)	92 (39.66)	76 (43.93)	0.688
Government/public	220 (54.32)	130 (56.03)	90 (52.02)	
Other	17 (4.20)	10 (4.31)	7 (4.05)	
Childhood Opportunity Index, overall				
Very low/low	180 (43.17)	118 (49.79)	62 (34.44)	0.004
Moderate	82 (19.66)	37 (15.61)	45 (25.00)	
High/very high	155 (37.17)	82 (34.60)	73 (40.56)	
Clinical characteristics				
Male	219 (52.52)	125 (52.74)	94 (52.22)	0.926
Prematurity (<37 wk)	90 (21.58)	61 (25.74)	29 (16.11)	0.018
Genetic diagnosis (yes)	131 (31.41)	81 (34.18)	50 (27.78)	0.315
DiGeorge (yes)	47 (11.27)	34 (14.35)	13 (7.22)	0.037
Prenatal diagnosis				
Yes	245 (58.75)	139 (58.65)	106 (58.89)	0.915
No	164 (39.33)	94 (39.66)	70 (38.89)	
Unknown	8 (1.92)	4 (1.69)	4 (2.22)	
Intubated prior to initial intervention	97 (23.26)	67 (28.27)	30 (16.67)	0.006
Anatomic diagnosis				
TOF/PS	207 (49.64)	123 (51.90)	84 (46.67)	0.290
TOF/PA	210 (50.36)	114 (48.10)	96 (53.33)	
CCRC center				
1	32 (7.67)	26 (10.97)	6 (3.33)	<0.001
2	110 (26.38)	37 (15.61)	73 (40.56)	
3	19 (4.56)	15 (6.33)	4 (2.22)	
4	68 (16.31)	59 (24.89)	9 (5.00)	
5	15 (3.60)	1 (0.42)	14 (7.78)	
6	49 (11.75)	35 (14.77)	14 (7.78)	
7	43 (10.31)	27 (11.39)	16 (8.89)	
8	32 (7.67)	13 (5.49)	19 (10.56)	
9	49 (11.75)	24 (10.13)	25 (13.89)	
Total	417	237	180	

Baseline maternal, socioeconomic, and clinical characteristics of neonates with symptomatic tetralogy of Fallot (sTOF), overall and stratified by staged repair vs primary complete repair. Data are presented as number (percentage). *P* values reflect univariate comparisons between management strategy groups using chi-square or Fisher exact tests, as appropriate. Childhood Opportunity Index (COI) reflects a composite neighborhood-level measure of educational, health, environmental, and socioeconomic resources.

CCRC = Congenital Cardiac Research Collaborative; TOF/PA = tetralogy of Fallot with pulmonary atresia; TOF/PS = tetralogy of Fallot with pulmonary stenosis.

### Primary outcomes

#### Associations between SDOH and management strategy

##### Unadjusted analysis

SDOH impact on the odds of undergoing SR was notable for children of non-Hispanic Black mothers who had a ∼2.3 times higher odds of undergoing a SR compared to non-Hispanic White individuals (*P* = 0.013). Individuals with a low or very low COI had ∼1.7 times higher odds of being in the SR group compared to those with a high/very high COI (*P* = 0.019). However, these differences did not remain statistically significant after adjustment for potential confounders (Table 2).

**Table 2:** Multivariable Logistic Regression Analysis of Social Drivers of Health Impact on Odds Ratio of a Staged Repair

	Unadjusted				Adjusted			
	OR	95% CI		*P* Value	OR	95% CI		*P* Value
Maternal characteristics								
Race-ethnicity								
Non-Hispanic Black vs non-Hispanic White	2.28	1.19	4.37	0.013	1.51	0.72	3.16	0.272
Hispanic vs non-Hispanic White	1.67	0.96	2.93	0.072	1.19	0.59	2.39	0.633
Primary language								
Spanish vs English	2.12	0.92	4.89	0.077	2.14	0.82	5.60	0.121
Socioeconomic characteristics								
Distance to primary cardiac center (miles)								
50-100 vs <50	1.06	0.67	1.67	0.807	1.56	0.91	2.70	0.109
>100 vs <50	0.98	0.60	1.59	0.929	0.67	0.37	1.22	0.185
Insurance								
Government/public vs private	1.19	0.80	1.79	0.393	0.80	0.49	1.30	0.367
Other insurance vs private	1.18	0.43	3.25	0.749	0.69	0.23	2.13	0.523
Childhood Opportunity Index								
Very low/low vs high/very high	1.69	1.09	2.63	0.019	0.97	0.57	1.63	0.897
Moderate vs high/very high	0.73	0.43	1.25	0.255	0.62	0.33	1.17	0.137

This table presents the results of the logistic regression analysis examining the impact of social drivers of health (SDOH) on repair strategy selection (primary repair vs staged repair). Primary repair is set as the reference group. The analysis provides both unadjusted and adjusted ORs, along with 95% CIs and *P* values. The adjusted model accounts for prematurity, DiGeorge syndrome, center, anatomical diagnosis, and intubation status.

##### Adjusted analyses

Clinical covariates from the fully adjusted management strategy model are shown in Supplemental Table 5. Prematurity and preoperative intubation showed nonsignificant trends toward SR, while center remained strongly associated with management strategy.

Our interaction analysis demonstrated a borderline association between COI and the increased likelihood of a SR strategy, which varied by maternal race-ethnicity (Race×COI global interaction, *P* = 0.057), as shown in Supplemental Table 6. After Bonferroni correction for multiple comparisons across race strata, this association remained significant (adjusted *P* = 0.048). Given this finding, the impact of COI within each race category was further explored. We found that among Hispanic patients, low COI was associated with higher odds of SR compared with high COI (OR: 6.59; 95% CI: 1.42-30.55; *P* = 0.016).

### Secondary outcomes

#### Associations between SDOH and mortality

Individual and composite SDOH measures by mortality are presented in a multivariable regression model in Table 3. In this cohort, there was an overall mortality of 10% at a median follow-up of 4.8 years (25th–75th %ile: 2.1-8.2). Patients living over 100 miles from a primary heart center had a 2.4x higher hazard for mortality than those living within a 50-mile radius (HR: 2.38; 95% CI: (1.07-5.30); *P* = 0.033). Maternal race-ethnicity, insurance, and COI were not associated with mortality. No association was observed between SDOH stratified by repair strategy and mortality. Clinical covariates included in the fully adjusted survival model for key exposures of interest, including race-ethnicity and COI, are shown in Supplemental Table 7, where preoperative intubation was associated with increased mortality, while other covariates, including center, were not significant.

**Table 3:** Multivariable Survival Analysis of Social Drivers of Health

	Unadjusted				Adjusted			
	HR	95% CI		*P* Value	HR	95% CI		*P* Value
Maternal characteristics								
Race-ethnicity								
Non-Hispanic Black vs non-Hispanic White	2.33	0.92	5.92	0.075	1.80	0.68	4.77	0.234
Hispanic vs non-Hispanic White	1.27	0.45	3.61	0.651	0.86	0.27	2.72	0.792
Socioeconomic characteristics								
Distance to primary heart center (miles)								
50-100 vs <50	1.14	0.52	2.48	0.745	1.50	0.65	3.47	0.341
>100 vs <50	2.20	1.07	4.51	0.031	2.38	1.07	5.30	0.033
Maternal insurance								
Government/public vs private	1.97	0.91	4.26	0.084	1.44	0.65	3.17	0.365
Other insurance vs private	2.31	0.50	10.67	0.285	2.00	0.42	9.48	0.384
Childhood Opportunity Index								
Very low/low vs high/very high	0.95	0.48	1.88	0.883	0.84	0.41	1.75	0.648
Moderate vs high/very high	0.94	0.40	2.20	0.888	0.96	0.39	2.33	0.925

This table presents the results of a multivariable survival analysis examining the impact of social drivers of health (SDOH) on survival. The analysis provides unadjusted and adjusted HRs, along with 95% CI and *P* values. The adjusted model accounts for prematurity, DiGeorge syndrome, center, anatomical diagnosis, and intubation status, and is stratified by repair strategy.

#### Associations between SDOH and total hospital LOS

**Figure 1:**
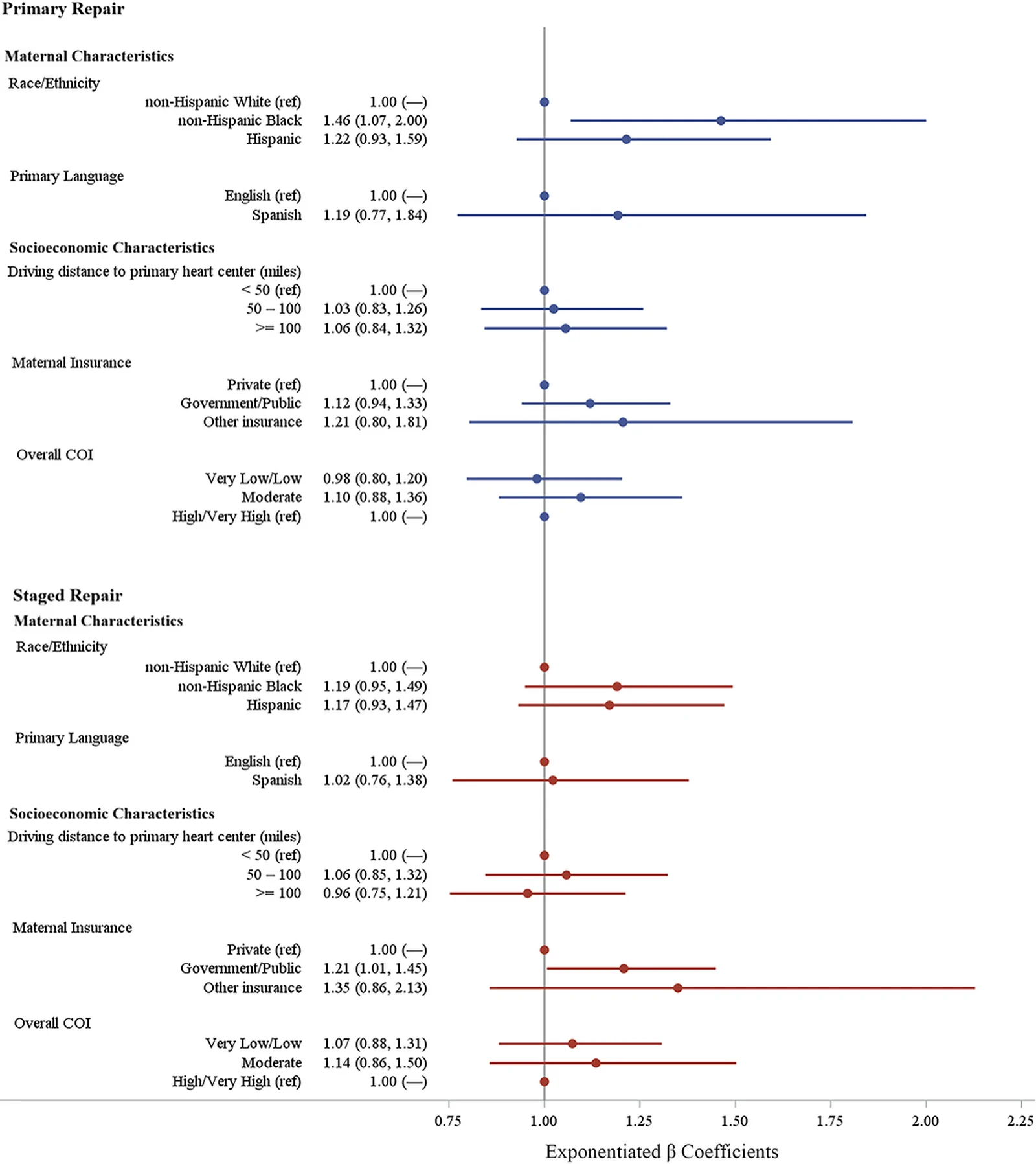
Forest Plot of Adjusted Exponentiated Regression Coefficients for Length of Stay by Social Drivers of Health Factors (eˆβ Estimates, 95% CIs) Results are derived from separate multivariable linear regression models. They are adjusted for prematurity, DiGeorge syndrome, center, anatomic diagnosis, intubation, and initial hospital complications, as well as stratified by repair strategy. Outcome values underwent logarithmic and exponential transformations for analysis. Race-ethnicity and government insurance were associated with increased length of stay. COI = Childhood Opportunity Index.

In adjusted multivariable analyses, among those undergoing PR, non-Hispanic Black infants experienced a 46% longer total hospital LOS than non-Hispanic White infants (Figure 1). In those who underwent an SR, infants with public insurance experienced a 21% longer total LOS than those with private insurance.

#### Association between SDOH and hospital complications

**Figure 2:**
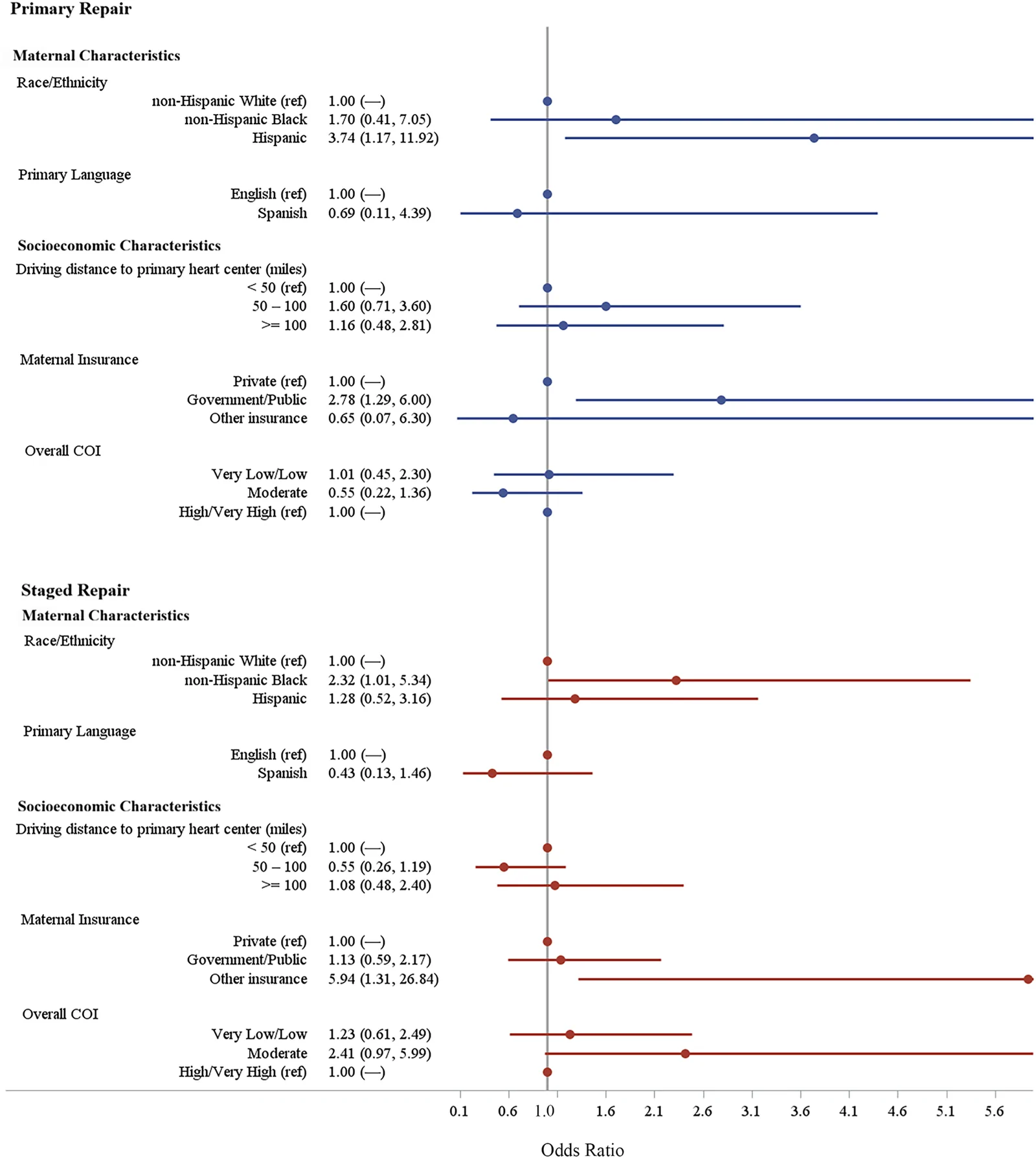
Forest Plot of Adjusted ORs for Hospital Complications by Social Drivers of Health Factors (ORs, 95% CIs) Results derived from separate multivariable logistic regression models, adjusted for prematurity, DiGeorge syndrome, center, anatomic diagnosis, and intubation, and stratified by repair strategy. Race-ethnicity and government and other insurance were associated with increased hospital complications. Abbreviation as in Figure 1.

On multivariable analysis of PR infants, the odds of having hospital complications was 274% higher for Hispanics compared to non-Hispanic Whites (Figure 2). Publicly insured infants also had a 178% higher odds of hospital complications compared to infants insured with private health insurance. Among those who underwent an SR, the odds of hospital complications was 132% higher among non-Hispanic Black compared to non-Hispanic White infants and 494% higher in those with “other” types of insurance as compared to privately insured patients.

## Discussion

This multicenter cohort study compared the impact of individual and composite neighborhood SDOH on initial management strategy, as well as mortality and morbidity, in neonates with sTOF. While COI and maternal race-ethnicity were found to be associated with the choice of PR vs SR in unadjusted analyses, these associations attenuated after multivariable adjustment (Central Illustration). In secondary outcome analyses, multiple SDOH measures, including COI, maternal race-ethnicity, insurance status, and geographic distance from a cardiac center, were all associated with morbidity and mortality for both PR and SR. Finally, in an exploratory analysis, although the global interaction term did not reach conventional statistical significance (*P* = 0.057), stratified analyses demonstrated that Hispanic infants from lower COI neighborhoods had higher odds of undergoing SR compared with those from higher COI areas.

**Central Illustration:**
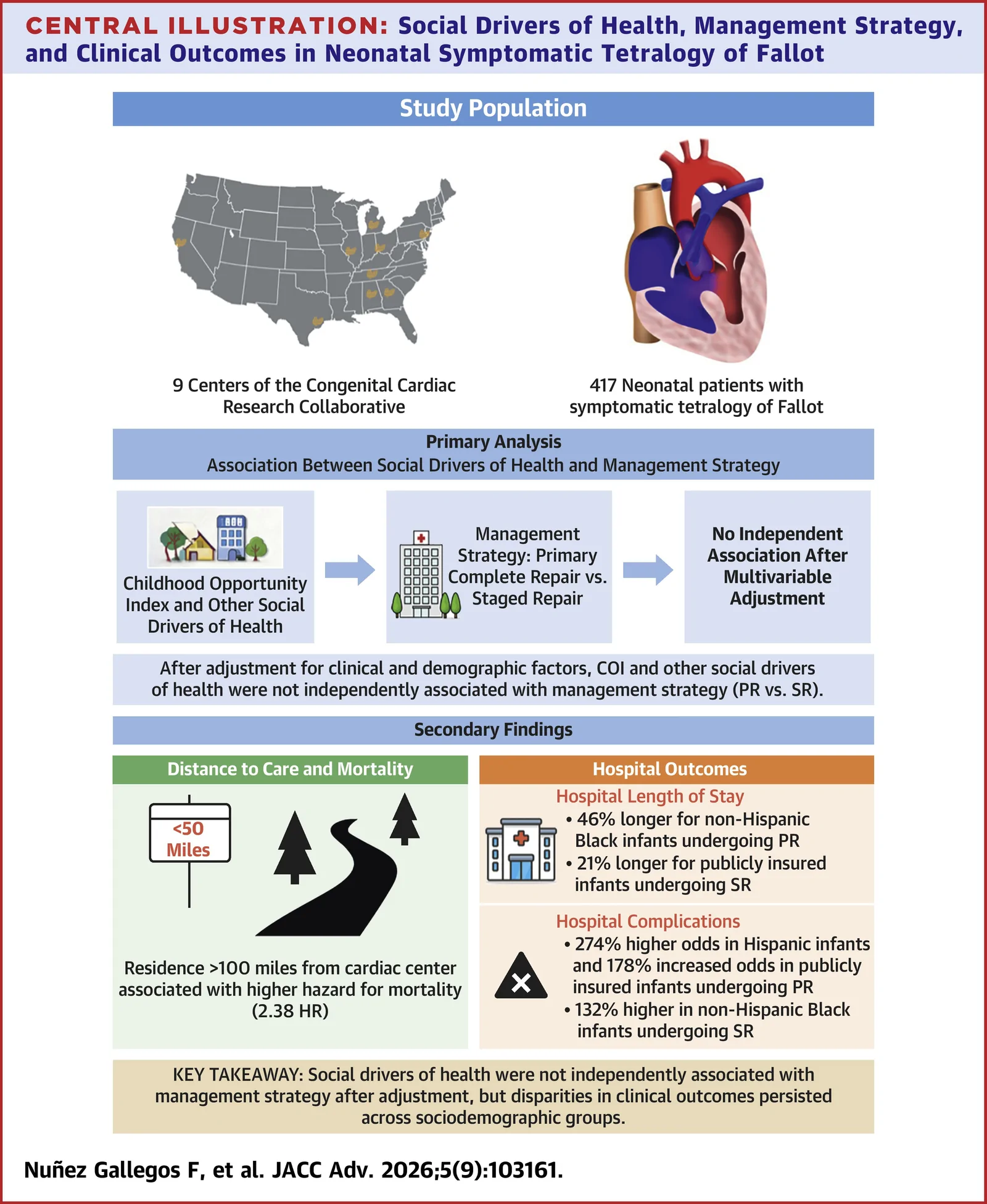
Social Drivers of Health, Management Strategy, and Clinical Outcomes in Neonatal Symptomatic Tetralogy of Fallot In a multicenter retrospective cohort study of 417 neonates with symptomatic tetralogy of Fallot, Childhood Opportunity Index, and other social drivers of health were evaluated in relation to management strategy and clinical outcomes. Although Childhood Opportunity Index and maternal race-ethnicity were associated with repair strategy in unadjusted analyses, these associations were not independently associated with management strategy after multivariable adjustment. In contrast, disparities in clinical outcomes persisted: residence >100 miles from a cardiac center was associated with a higher hazard of mortality, while maternal race-ethnicity and insurance status were associated with differences in hospital length of stay and complication rates. PR = primary complete repair; SR = staged repair; other abbreviation as in Figure 1.

Our data revealed that while SDOH may not independently determine the initial surgical approach, they remain strongly associated with clinical outcomes and the broader care experience for neonates with sTOF. Thus, structural and SDOH factors in neonates with sTOF continue to influence both decision-making and outcomes in highly specialized congenital cardiac care. Furthermore, the impact of SDOH on outcomes in sTOF is not confined to broad, well-recognized disparities; rather, specific, clinically actionable points within the care continuum are where inequities clearly emerge.

Inequities also arise at other modifiable points along the care pathway as demonstrated by the persistence of disparities in mortality, hospital LOS, and complications even after accounting for repair strategy. Access to high-quality prenatal care, timeliness of diagnosis, peri-operative support, availability of social resources during hospitalization, and distance-related barriers to follow-up may play a significant role. From a public health perspective, these patterns suggest actionable targets, including standardizing prenatal counseling and surgical decision-making processes, strengthening care coordination for families facing socioeconomic disadvantage or geographic isolation, and integrating equity-oriented supports throughout preoperative, peri-operative, and postoperative care. Together, these interventions represent opportunities to reduce inequities and improve outcomes for all infants with sTOF.

### SDOH and strategy selection (primary outcome)

Associations between COI, maternal race/ethnicity, and intervention type were attenuated after adjusting for covariates, underscoring the complexity of disentangling social from clinical influences, as management strategy is multifactorial and not guided by standardized anatomical criteria and may also reflect center- and provider-level preferences. This raises the critical question of why such differences in strategy selection occur and whether they reflect differential communication, variation in prenatal counseling, unmeasured clinical nuance, or reliance on clinical heuristics shaped by structural inequity.

Our adjusted interaction analysis suggested a potential differential association between COI and repair strategy among Hispanic patients (*P* = 0.057). Stratified analyses indicated that Hispanic infants from lower COI neighborhoods had higher odds of undergoing SR compared with those from higher COI areas. Thus, management strategy may function not merely as a covariate to be adjusted away but potentially as a mechanistic contributor to downstream disparities, particularly when the choice between PR and SR varies systematically by maternal race-ethnicity and COI. When substantial practice variation intersects with uncertain, highly complex outcome data, clinicians may rely on heuristics to guide their decisions. However, these shortcuts can inadvertently reflect implicit bias or underlying structural inequities, resulting in unequal treatment across patient groups. These dynamics also pose methodological challenges, as the influence of social and structural determinants on provider decision-making can be difficult to isolate. Moreover, research in pediatric settings more broadly shows that providers may, often unconsciously, tailor management recommendations based on perceived patient social support, health literacy, or adherence potential, which may disadvantage lower-SES families.^16^^,^^17^ In children undergoing CHD interventions, these dynamics may contribute to differing surgical strategies or timing beyond strictly anatomical or physiologic considerations.

### Secondary outcomes

#### SDOH and sTOF morbidity

We found persistent disparities in hospital LOS and complications in minoritized infants and in those with public or “other” insurance, even after adjusting for clinical risk factors. These findings are consistent with previous studies in CHD populations, where minoritized racial groups and government-insured patients have been shown to experience variations in LOS and time to intervention.^10^^,^^18^^,^^19^ These disparities may stem from differences in discharge planning, care coordination, and communication between families and care teams. Prior literature suggests that racially discordant care is associated with less shared decision-making and lower patient engagement, potentially influencing perceptions of family readiness for discharge.^20^, ^21^, ^22^, ^23^ Discrepant outcomes may also reflect implicit provider bias, differences in health literacy, or challenges in securing follow-up care and services. Importantly, our study calculated LOS outside of the immediate peri-operative window to reduce bias related to procedural complexity. Thus, increased LOS among minoritized groups likely reflects postoperative and systems-level challenges, not surgical delay or intraoperative complications.

#### Neighborhood COI and composite SDOH measures

While prior studies have linked COI to poor outcomes in pediatric and CHD populations,^7^^,^^24^^,^^25^ we found that composite measures, such as the COI, were not independently associated with morbidity or mortality, although they were associated with the initial management strategy. This suggests that individual-level SDOH markers may exert stronger or more direct influences on medical decision-making, particularly in highly medicalized, acute care contexts. Furthermore, composite indices may obscure meaningful variation or be overcorrected in multivariable models, masking their true effect. These results reinforce the need to use both community and individual-level SDOH measures to understand the origins and mechanisms of health disparities.

#### Impact of geography on mortality

In line with prior literature, our study highlights the vital role of geography and distance in achieving equitable outcomes in CHD.^26^ Distance to care likely reflects both geographic access and patterns of regionalized referral, which are not fully captured in this data set. Similar findings of worse outcomes among those living further from a cardiac center were found among infants with hypoplastic left heart syndrome^3^ and in CHD infant mortality based on proximity to a metropolitan area with a top-ranked pediatric cardiac center.^27^ Furthermore, geography-related mortality risk reflects broader challenges within the fragmented U.S. health care system, such as transportation, housing, and continuity of care, where those with the least flexible resources must often expend the most to access subspecialty care.^28^^,^^29^ Data show that families with higher SES can more easily mobilize resources to obtain care at high-performing hospitals, irrespective of distance.^30^ In comparison, those with lower SES face economic, social, and logistical burdens that may compound over time.^29^ These lower SES individuals are forced to tap into more resources to travel a longer distance to obtain care, causing economic and social stressors on families (opportunity costs such as missed days of work and income), which may be a critical factor in the mortality risk of these infants. Collectively, these findings reflect the real-world impact of geographic health inequity and may suggest that centralized or regionalized^31^^,^^32^ congenital cardiac care may unintentionally introduce barriers to access that impact long-term survival and that stabilization in tiered “essential” specialized care centers with access to comprehensive care institutions for more complex CHD may assist in reducing distance disparities.^33^

#### Implications for health equity in CHD

Our findings using the CCRC cohort offer a unique perspective: participating centers are peer institutions with comparable expertise, resources, and subspecialty teams. Because these demonstrate that while infants were treated at similarly resourced centers, measured differences are less likely to reflect variation in institutional capability and more likely to reflect factors operating within institutions or across shared care processes. The racial and socioeconomic disparities observed in our study must be viewed within the context of structural racism and unequal distribution of health care resources.^34^, ^35^, ^36^, ^37^ The unequal burden of morbidity borne by minoritized and low-SES populations highlights the need for equity-centered quality improvement initiatives across pediatric cardiac programs.

Future efforts should aim to understand the interplay among patient-level SDOH, COI, and access to specialized cardiac care and how these factors intersect with institutional practices to shape decision-making and outcomes by: 1) standardizing surgical decision-making; 2) improving access to prenatal diagnosis and coordinated care pathways; and 3) implementing culturally responsive communication strategies. Investigating referral patterns, center volume effects, and prenatal counseling disparities will be crucial in identifying upstream drivers of inequity that persist even among peer centers.

### Study limitations

In addition to those already addressed, we acknowledge the following limitations. As a retrospective cohort analysis, causal inference is limited, and unmeasured confounding remains a potential issue. Although formal mediation analyses did not identify significant indirect effects, some covariates—particularly prematurity and DiGeorge syndrome in the management strategy models and hospital complications in the LOS models—may function as partial mediators in certain causal frameworks. Accordingly, adjustment for these variables may have introduced overadjustment bias, potentially attenuating the observed associations between SDOH and the outcomes of interest. This represents an inherent limitation of observational studies with complex causal pathways. While COI provides valuable insight into neighborhood-level SDOH, more granular individual-level measures of SES (eg, income, education, and wealth) were unavailable, and detailed procedural characteristics and outcomes were the focus of prior CCRC work on which this study builds.^5^ In addition, maternal race-ethnicity was self-reported, and we were unable to disaggregate outcomes for participants categorized as “other,” which likely includes individuals with diverse racial and ethnic backgrounds.

Although we used the address at the time of index hospitalization to calculate COI and distance, families may have moved during the study period. However, prior research has shown that COI scores remain relatively stable even in families that move due to persistent neighborhood-level inequities.^38^ Additionally, our findings may not be generalizable to lower-volume centers, as the CCRC includes primarily medium- and high-volume institutions with strong overall outcomes.

While our interaction analysis suggested a borderline association between COI and the likelihood of a SR strategy that varied by maternal race/ethnicity, we emphasize that these analyses are exploratory; the borderline global interaction, along with wide confidence intervals, raises the possibility that this finding may represent a chance association. As such, this result should be considered hypothesis-generating and requires confirmation in future studies.

Finally, while these analyses were not corrected for multiple comparisons and may be subject to residual confounding, the secondary findings highlight meaningful patterns in how social and structural factors relate to care processes and outcomes in this population.

## Conclusions

In a large multicenter cohort of neonatal infants with sTOF, neighborhood opportunity, race-ethnicity, insurance type, and distance to care are associated with postoperative morbidity, and distance to care independently predicts mortality. These findings highlight the importance of measuring and addressing both individual- and system-level inequities in the delivery of CHD care. Moving forward, equity-focused interventions, improved access to specialized care, and a deeper understanding of how families navigate the health care system are essential to reducing disparities in CHD outcomes.

Perspectives **COMPETENCY IN SYSTEMS-BASED PRACTICE:** In neonates with symptomatic tetralogy of Fallot, neighborhood opportunity and maternal race-ethnicity were not independently associated with initial management strategies once covariates were incorporated into the models. However, maternal race-ethnicity and insurance were associated with longer hospital LOS, and greater geographic distance to a congenital heart center is associated with increased long-term mortality, highlighting the clinical impact of social and structural factors on outcomes.

**TRANSLATIONAL OUTLOOK:** Future research and quality-improvement initiatives should integrate both neighborhood-level and individual sociodemographic measures to better identify and address inequities in the management and outcomes of infants with symptomatic tetralogy of Fallot. Equity-focused interventions targeting surgical decision-making, postoperative morbidity, and access to specialized care, particularly for families facing geographic and structural barriers, may reduce disparities in survival and advance equitable CHD care.

## Funding support and author disclosures

This study was partly funded by generous support from the member institutions of the CCRC, The Liam Sexton Foundation, A Heart Like Ava, and the Kennedy Hammill Pediatric Cardiac Research Fund. Dr Qureshi is a consultant for W.L Gore and Associates, Medtronic Inc, Abiomed Inc, and B. Braun. All other authors have reported that they have no relationships relevant to the contents of this paper to disclose.

## References

[1] Oster M.E., Strickland M.J., Mahle W.T. (2011). Racial and ethnic disparities in post-operative mortality following congenital heart surgery. J Pediatr.

[2] Lopez K.N., Morris S.A., Sexson Tejtel S.K., Espaillat A., Salemi J.L. (2020). US mortality attributable to congenital heart disease across the lifespan from 1999 through 2017 exposes persistent racial/ethnic disparities. Circulation.

[3] Morris S.A., Ethen M.K., Penny D.J. (2014). Prenatal diagnosis, birth location, surgical center, and neonatal mortality in infants with hypoplastic left heart syndrome. Circulation.

[4] Lara D.A., Fixler D.E., Ethen M.K., Canfield M.A., Nembhard W.N., Morris S.A. (2016). Prenatal diagnosis, hospital characteristics, and mortality in transposition of the great arteries. Birt Defects Res A Clin Mol Teratol.

[5] Goldstein B.H., Petit C.J., Qureshi A.M. (2021). Comparison of management strategies for neonates with symptomatic tetralogy of fallot. J Am Coll Cardiol.

[6] O’Byrne M.L., Glatz A.C., Huang Y.V. (2022). Comparative costs of management strategies for neonates with symptomatic tetralogy of fallot. J Am Coll Cardiol.

[7] Duong S.Q., Elfituri M.O., Zaniletti I. (2023). Neighborhood childhood opportunity, race/ethnicity, and surgical outcomes in children with congenital heart disease. J Am Coll Cardiol.

[8] Sengupta A., Gauvreau K., Bucholz E.M., Newburger J.W., del Nido P.J., Nathan M. (2022). Contemporary socioeconomic and childhood opportunity disparities in congenital heart surgery. Circulation.

[9] Peterson J.K., Chen Y., Nguyen D.V., Setty S.P. (2017). Current trends in racial, ethnic, and healthcare disparities associated with pediatric cardiac surgery outcomes. Congenit Heart Dis.

[10] Anderson B.R., Fieldston E.S., Newburger J.W., Bacha E.A., Glied S.A. (2018). Disparities in outcomes and resource use after hospitalization for cardiac surgery by neighborhood income. Pediatrics.

[11] Best K.E., Vieira R., Glinianaia S.V., Rankin J. (2019). Socio-economic inequalities in mortality in children with congenital heart disease: a systematic review and meta-analysis. Paediatr Perinat Epidemiol.

[12] Law M.A., Glatz A.C., Romano J.C. (2022). Palliation strategy to achieve complete repair in symptomatic neonates with tetralogy of fallot. Pediatr Cardiol.

[13] Census geocoder. https://geocoding.geo.census.gov/geocoder/geographies/onelineaddress?form.

[14] Zaidi A.H., Alberts A., Chowdhury D. (2024). Trends in gaps of care for patients with congenital heart disease: implications for social determinants of health and child opportunity index. J Am Heart Assoc Cardiovasc Cerebrovasc Dis.

[15] Welke K.F., Pasquali S.K., Lin P. (2019). Hospital distribution and patient travel patterns for congenital cardiac surgery in the United States. Ann Thorac Surg.

[16] Addala A., Hanes S., Naranjo D., Maahs D.M., Hood K.K. (2021). Provider implicit bias impacts pediatric type 1 diabetes technology recommendations in the United States: findings from the gatekeeper study. J Diabetes Sci Technol.

[17] Sabin J.A., Greenwald A.G. (2012). The influence of implicit bias on treatment recommendations for 4 common pediatric conditions: pain, urinary tract infection, attention deficit hyperactivity disorder, and asthma. Am J Public Health.

[18] DiBardino D.J., Pasquali S.K., Hirsch J.C. (2012). Effect of sex and race on outcome in patients undergoing congenital heart surgery: an analysis of the society of thoracic surgeons congenital heart surgery database. Ann Thorac Surg.

[19] Milazzo A.S., Sanders S.P., Armstrong B.E., Li J.S. (2002). Racial and geographic disparities in timing of bidirectional glenn and fontan stages of single-ventricle palliation. J Natl Med Assoc.

[20] Beach M.C., Saha S., Korthuis P.T. (2011). Patient–provider communication differs for black compared to white HIV-infected patients. AIDS Behav.

[21] Beach M.C., Saha S., Korthuis P.T. (2010). Differences in patient–provider communication for Hispanic compared to non-hispanic white patients in HIV care. J Gen Intern Med.

[22] Rodriguez H.P., von Glahn T., Grembowski D.E., Rogers W.H., Safran D.G. (2008). Physician effects on racial and ethnic disparities in patients’ experiences of primary care. J Gen Intern Med.

[23] Shen M.J., Peterson E.B., Costas-Muñiz R. (2017). The effects of race and racial concordance on patient-physician communication: a systematic review of the literature. J Racial Ethn Health Disparities.

[24] Garg A., Sochet A.A., Hernandez R., Stockwell D.C. (2024). Association of the child opportunity index and inpatient illness severity in the United States, 2018–2019. Acad Pediatr.

[25] Mayourian J., Brown E., Javalkar K. (2023). Insight into the role of the child opportunity index on surgical outcomes in congenital heart disease. J Pediatr.

[26] Bebbington M. (2022). Barriers to accessing care: challenges in early prenatal diagnosis of congenital anomalies. Ultrasound Obstet Gynecol.

[27] Kaltman J.R., Burns K.M., Pearson G.D., Goff D.C., Evans F. (2020). Disparities in congenital heart disease mortality based on proximity to a specialized pediatric cardiac center. Circulation.

[28] Barnea E.R., Nicholson W., Theron G. (2021). From fragmented levels of care to integrated health care: framework toward improved maternal and newborn health. Int J Gynecol Obstet.

[29] Phelan J.C., Link B.G., Tehranifar P. (2010). Social conditions as fundamental causes of health inequalities: theory, evidence, and policy implications. J Health Soc Behav.

[30] Link B.G., Phelan J. (1995). Social conditions as fundamental causes of disease. J Health Soc Behav.

[31] Welke K.F., Pasquali S.K., Lin P. (2020). Regionalization of congenital heart surgery in the United States. Semin Thorac Cardiovasc Surg.

[32] Burki S., Fraser C.D. (2016). Larger centers may produce better outcomes: is regionalization in congenital heart surgery a superior model?. Semin Thorac Cardiovasc Surg Pediatr Card Surg Annu.

[33] Backer C.L., Overman D.M., Dearani J.A. (2023). Recommendations for centers performing pediatric heart surgery in the United States. J Thorac Cardiovasc Surg.

[34] Huang S.J., Sehgal N.J. (2022). Association of historic redlining and present-day health in Baltimore. PLoS One.

[35] Paquette E.T. (2022). Reckoning with redlining and other structural barriers to health of critically ill children: addressing systemic racism will require shifting the focus from Micro- to macrolevel analysis of social risks. Pediatr Crit Care Med.

[36] Koh H.K. (2011). The ultimate measures of health. Public Health Rep.

[37] (2019). Racism: science & tools for the public health professional.

[38] Root E.D., Humphrey J.L. (2014). The impact of childhood mobility on exposure to neighborhood socioeconomic context over time. Am J Public Health.

