# Supplementary material for: Social Drivers of Health, Management Bias, and Outcomes of Symptomatic Neonates With Tetralogy of Fallot
Source: JACC Adv. 2026 Aug 20;5(9):103161. doi: 10.1016/j.jacadv.2026.103161 (PMC13524577; doi:10.1016/j.jacadv.2026.103161)
Supplement: Supplementary file 1 [file mmc1.docx]

**Supplemental Table 1:** Congenital Cardiac Research Collaborative Participating Centers

Cincinnati Children’s Hospital, Cincinnati, OH

Children’s Hospital of Alabama, Birmingham, AL

*Children’s Hospital of Atlanta, Atlanta, GA

Children’s Hospital of Philadelphia, Philadelphia, PA

C.S. Mott Children’s Hospital, Ann Arbor, MI

Monroe Carell Jr Children’s Hospital at Vanderbilt, Nashville, TN

St. Louis Children’s Hospital, St. Louis, MO

Texas Children’s Hospital, Houston, TX

UCSF Benioff Children’s Hospital, San Francisco, CA

^*Collaborative Data Coordinator Center^

**Supplemental Table 1 lists the** participating centers in the Congenital Cardiac Research Collaborative, including nine tertiary pediatric cardiac centers across the United States, with the coordinating data center indicated.

**Supplemental Table 2:** Hospital Complications

Acute kidney injury

Cardiac arrest

Aspiration

Arrhythmia

Coronary artery injury

Death

Dialysis

Extracorporeal Membrane Oxygenation

Gastrointestinal pathology

Hemidiaphragm paresis

Hypoglycemia

Infection

Necrotizing enterocolitis

Neurological injury

Pericardial tamponade

Pulmonary hemorrhage

Surgical bleeding

Thrombosis

Unplanned reintervention

Vascular complication

Vocal cord paresis

Supplemental Table 2 details the hospital complications included in the composite morbidity outcome, encompassing cardiovascular, respiratory, neurologic, infectious, and procedural adverse events.

**Supplemental Table 3:** Additional Clinical and Anatomic Characteristics of the Study Cohort and Initial Palliative Procedures Among Infants Undergoing Staged Repair

|  | **Overall**  **N = 417** | **Staged Repair**  **n = 237 (57%)** | **Primary Repair**  **n = 180 (43%)** | **p-value** |
| --- | --- | --- | --- | --- |
| **Clinical and Anatomic Characteristics** | | | | |
| Gestational age, weeks, median (IQR) | 38.4 (37.0, 39.4) | 38.1 (36.9, 39.3) | 39.0 (37.0, 39.6) | **0.005** |
| Age at index procedure, days, median (IQR) | 7 (5, 15) | 7 (5, 13) | 7 (4, 17) | 0.40 |
| Weight at index procedure, kg, median (IQR) | 2.9 (2.5, 3.4) | 2.9 (2.5, 3.3) | 3.0 (2.6, 3.5) | **0.046** |
| Right pulmonary artery diameter, mm, median (IQR) | 3.7 (3.2, 4.2) | 3.6 (3.2, 4.1) | 3.8 (3.2, 4.3) | 0.19 |
| Right pulmonary artery z-score, median (IQR) | −1.41 (−1.93, −0.90) | −1.45 (−1.92, −0.90) | −1.40 (−1.93, −0.80) | 0.64 |
| Left pulmonary artery diameter, mm, median (IQR) | 3.5 (3.0, 4.0) | 3.5 (2.9, 4.0) | 3.5 (3.0, 4.0) | **0.037** |
| Left pulmonary artery z-score, median (IQR) | −1.42 (−1.93, −0.89) | −1.51 (−2.02, −0.92) | −1.34 (−1.83, −0.87) | 0.24 |
| Inotrope use before index procedure | 49 (11.78) | 32 (13.56) | 17 (9.44) | 0.197 |
| Initial Form of Palliation | | | | |
| RVOT stent | 8 (3.4%) | 8 (3.4%) | N/A |  |
| PDA stent | 16 (6.8%) | 16 (6.8%) | N/A |  |
| Balloon pulmonary valvuloplasty (BPV) | 30 (12.7%) | 30 (12.7%) | N/A |  |
| Surgical shunt (modified BTT shunt) | 174 (73.4%) | 174 (73.4%) | N/A |  |
| Surgical RVOT patch (without VSD closure) | 9 (3.8%) | 9 (3.8%) | N/A |  |

Supplemental Table 3. Additional clinical and anatomic characteristics by management strategy and initial palliative procedures among infants undergoing staged repair. Data are presented as median (IQR) or n (%). Initial palliative procedures are shown for the staged repair group only.

**Supplemental Table 4:** Comparison of baseline demographic characteristics, clinical characteristics, and clinical outcomes between patients with and without available Childhood Opportunity Index (COI) data.

|  | **Included patients (COI available)** | **Excluded patients (COI unavailable)** | **p-value*** |
| --- | --- | --- | --- |
|  | **N = 417** | **N = 155** |  |
|  | **N (%)** | **N (%)** |  |
| **Maternal Characteristics** |  |  |  |
| Race-ethnicity |  |  |  |
| non-Hispanic White | 215 (63.05) | 86 (67.72) | 0.461 |
| non-Hispanic Black | 55 (16.13) | 21 (16.54) |  |
| Hispanic | 71 (20.82) | 20 (15.75) |  |
| **Clinical Characteristics** |  |  |  |
| Male Sex | 219 (52.52) | 88 (56.77) | 0.364 |
| Prematurity (<37 weeks) | 90 (21.58) | 25 (16.13) | 0.087 |
| Genetic Diagnosis (Yes) | 131 (31.41) | 45 (29.03) | 0.583 |
| DiGeorge (Yes) | 47 (11.27) | 15 (9.68) | 0.586 |
| Prenatal Diagnosis |  |  |  |
| Yes | 245 (58.75) | 70 (45.16) | **0.01** |
| No | 164 (39.33) | 79 (50.97) |  |
| Unknown | 8 (1.92) | 6 (3.87) |  |
| Intubated prior to initial intervention | 97 (23.26) | 46 (29.68) | 0.064 |
| Anatomic Diagnosis |  |  |  |
| TOF/PS | 207 (49.64) | 77 (49.68) | 0.994 |
| TOF/PA | 210 (50.36) | 78 (50.32) |  |
| CCRC Center |  |  |  |
| 1 | 32 (7.67) | 18 (11.61) | **<0.001** |
| 2 | 110 (26.38) | 26 (16.77) |  |
| 3 | 19 (4.56) | 7 (4.52) |  |
| 4 | 68 (16.31) | 9 (5.81) |  |
| 5 | 15 (3.60) | 15 (9.68) |  |
| 6 | 49 (11.75) | 12 (7.74) |  |
| 7 | 43 (10.31) | 42 (27.10) |  |
| 8 | 32 (7.67) | 9 (5.81) |  |
| 9 | 49 (11.75) | 17 (10.97) |  |
| **Outcomes** |  |  |  |
| Hospital complication | 133 (31.89) | 34 (21.94) | **0.02** |
| Length of Stay | 27 (17–40) | 25 (16–42) | 0.965 |
| Mortality | 41 (9.83) | 11 (7.10) | 0.312 |

Supplemental Table 4 compares baseline demographic characteristics, clinical characteristics, and clinical outcomes between patients with and without available Childhood Opportunity Index (COI) data as a sensitivity analysis assessing potential selection bias. *P values were calculated using Pearson χ² or Fisher's exact test for categorical variables and the Wilcoxon rank-sum test for total hospital length of stay.

**Supplemental Table 5:** Multivariable Logistic Regression Analysis of Covariables

| Variable | HR (95% CI) | p-value |
| --- | --- | --- |
| Prematurity (Yes vs No) | 2.46 (1.27–4.79) | 0.08 |
| DiGeorge Syndrome (Yes vs No) | 1.80 (0.79–4.08) | 0.163 |
| Preoperative Intubation (Yes vs No) | 1.94 (1.00–3.79) | 0.051 |
| Anatomic Diagnosis (TOF/PA vs TOF/PS) | 0.82 (0.49–1.37) | 0.442 |
| Center (ref: Center 1) |  | <0.001 |
| Center 2 | 0.11 (0.03–0.34) | 0.0002 |
| Center 3 | 0.57 (0.12–2.73) | 0.477 |
| Center 4 | 0.95 (0.25–3.56) | 0.938 |
| Center 5 | 0.02 (0.00–0.18) | 0.0007 |
| Center 6 | 0.53 (0.15–1.91) | 0.33 |
| Center 7 | 0.24 (0.07–0.85) | 0.027 |
| Center 8 | 0.22 (0.04–1.14) | 0.07 |
| Center 9 | 0.18 (0.05–0.66) | 0.009 |

* Supplementary Table Related to Table 2

Supplemental Table 5 presents the clinical covariates included in the fully adjusted multivariable models (Table 2) used to evaluate key exposures of interest, including race-ethnicity and COI. Prematurity and preoperative intubation showed trends toward higher odds of staged repair but were not statistically significant. Center remained a strong independent predictor, with significant variation in management strategy across institutions.

**Supplemental Table 6:** Impact of COI within Race-ethnicity for Risk of Staged Repair

| **Section** | **Contrast** | **Adjusted OR (95% CI)** | **p-value** |
| --- | --- | --- | --- |
| **Global interaction**  **Bonferroni correction** | Race × COI (Type III joint test, df = 4) | — | 0.057  0.048 |
| **COI simple effects within race** | Very Low/Low vs High/Very High at Non-Hispanic White | 0.65 (0.30–1.41) | 0.272 |
|  | Moderate vs High/Very High at Non-Hispanic White | 0.56 (0.24–1.30) | 0.176 |
|  | Very Low/Low vs High/Very High at Non-Hispanic Black | 2.93 (0.58–14.83) | 0.193 |
|  | Moderate vs High/Very High at Non-Hispanic Black | 3.25 (0.30–34.96) | 0.331 |
|  | Very Low/Low vs High/Very High at Hispanic | **6.59 (1.42–30.55)** | **0.016** |
|  | Moderate vs High/Very High at Hispanic | 1.58 (0.25–10.18) | 0.631 |

**Supplemental Table 6.** Interaction analysis examining the effect of Childhood Opportunity Index (COI) within race-ethnicity on the odds of staged repair. A borderline significant global interaction was observed, with a significant association between low COI and increased odds of staged repair among Hispanic infants, while no significant associations were observed within non-Hispanic White or non-Hispanic Black groups.
**Supplemental Table 7:** Multivariable Survival Analysis of Covariables

| **Variable** | **HR (95% CI)** | **p-value** |
| --- | --- | --- |
| **Prematurity (Yes vs No)** | 2.13 (0.84–5.42) | 0.11 |
| **DiGeorge Syndrome (Yes vs No)** | 1.26 (0.41–3.84) | 0.69 |
| **Preoperative Intubation (Yes vs No)** | 3.08 (1.34–7.09) | 0.008 |
| **Anatomic Diagnosis (TOF/PA vs TOF/PS)** | 0.43 (0.18–1.02) | 0.055 |
| **Center** (ref: Center 1) |  | 0.714 |
| Center 2 | 0.48 (0.09–2.49) | 0.38 |
| Center 3 | 0.52 (0.05–5.15) | 0.57 |
| Center 4 | 1.10 (0.25–4.73) | 0.9 |
| Center 5 | — | — |
| Center 6 | 2.13 (0.51–8.97) | 0.3 |
| Center 7 | 0.81 (0.16–4.07) | 0.8 |
| Center 8 | — | — |
| Center 9 | 0.67 (0.11–4.09) | 0.66 |

* Supplementary Table Related to Table 3

Supplemental Table 7 presents the clinical covariates included in the fully adjusted multivariable survival models (Table 3) used to evaluate key exposures of interest, including race-ethnicity and COI. Preoperative intubation was significantly associated with increased mortality, while other covariates, including prematurity, DiGeorge syndrome, anatomic diagnosis, and center, were not significantly associated with survival.
